# Decreases in Young Children Who Received Blood Lead Level Testing During COVID-19 — 34 Jurisdictions, January–May *2*020

**DOI:** 10.15585/mmwr.mm7005a2

**Published:** 2021-02-05

**Authors:** Joseph G. Courtney, Stella O. Chuke, Kelly Dyke, Kimball Credle, Carolina Lecours, Kathryn B. Egan, Monica Leonard

**Affiliations:** 1 Division of Environmental Health Science and Practice, National Center for Environmental Health, CDC.

Exposure to lead, a toxic metal, can result in severe effects in children, including decreased ability to learn, permanent neurologic damage, organ failure, and death. CDC and other health care organizations recommend routine blood lead level (BLL) testing among children as part of well-child examinations to facilitate prompt identification of elevated BLL, eliminate source exposure, and provide medical and other services (*1*). To describe BLL testing trends among young children during the coronavirus disease 2019 (COVID-19) pandemic, CDC analyzed data reported from 34 state and local health departments about BLL testing among children aged <6 years conducted during January–May 2019 and January–May 2020. Compared with testing in 2019, testing during January–May 2020 decreased by 34%, with 480,172 fewer children tested. An estimated 9,603 children with elevated BLL were missed because of decreased BLL testing. Despite geographic variability, all health departments reported fewer children tested for BLL after the national COVID-19 emergency declaration (March–May 2020). In addition, health departments reported difficulty conducting medical follow-up and environmental investigations for children with elevated BLLs because of staffing shortages and constraints on home visits associated with the pandemic. Providers and public health agencies need to take action to ensure that children who missed their scheduled blood lead screening test, or who required follow-up on an earlier high BLL, be tested as soon as possible and receive appropriate care.

CDC identifies no safe BLL in children and considers a blood lead reference value (BLRV) of 5.0 *μ*g/dL* sufficient to prompt clinical and public health intervention (*1**,**2*). Among children aged <6 years, very high BLL (>70 *μ*g/dL) can cause neurologic problems (e.g., seizures or coma), organ failure, and death. Lower, but still elevated, BLL can affect the nervous system, causing permanent neurologic damage, behavioral disorders, and cognitive impairment (*1*). In the United States, the most common childhood lead exposures are from lead-based paint that was used in pre-1978 housing,^†^ lead-contaminated soil or lead-containing pollutants from industrial sources, and water from old lead pipes and fixtures (*3*). Very young children might ingest lead dust or paint because of their tendency to put fingers or objects (toys or paint chips) in their mouths, and they more readily absorb lead because their bodies are rapidly developing. Primary prevention focuses on reducing lead exposures in homes, schools, and communities. Secondary prevention consists of BLL screening as part of routine well-child examinations. Early identification of children with lead exposure can help identify and eliminate lead sources (and future exposures for other children); reduce their BLL over time; and link children with high BLLs to medical, nutritional, and educational services. Medicaid-enrolled children are required to be screened at ages 12 and 24 months; many states have additional screening requirements (*4*).

In 1995, elevated BLLs became a nationally reportable condition (*5*). CDC funds 53 state and local childhood lead poisoning prevention programs to conduct ongoing surveillance of BLL testing among children.^§ ^During May and June 2020, CDC received anecdotal reports of declines in BLL testing. To understand BLL testing trends during the COVID-19 pandemic, including after a national emergency was declared in March 2020, CDC requested that state and local health departments report the total number of children aged <6 years with BLL tests by month during January–May 2019 and January–May 2020. This activity was reviewed by CDC and was conducted consistent with applicable federal law and CDC policy.^¶^ Health departments could also submit qualitative information. Based on the 2007–2010 National Health and Nutrition Examination Survey (NHANES) data and subsequent trends** (*1*), an estimated 2.0% of children who did not have a BLL test were conservatively assumed to have levels exceeding the BLRV.

Data for the period of interest for children aged <6 years were received from 34 state and local health departments, including the District of Columbia and New York City.^†† ^Overall, the number of children aged <6 years who had BLL tests during January–May 2020 (948,844) was lower by 33.6% (480,172) than the number who had BLL tests during January–May 2019 (1,429,016) (Figure), resulting in an estimated 9,603 children with elevated BLLs being missed. During the analysis period, the number of children with BLL testing was lower during every month during January–May 2020 compared with the number with testing during the same period in 2019; the largest proportional decrease (66.4%) occurred in April 2020. During the early pandemic period (March–May 2020), the number of children with BLL tests (481,199) decreased by 52.5% compared with the same period in 2019 (880,812). Despite geographic variation, all 34 responding state and local health departments reported decreased BLL testing during March–May 2020 compared with testing during 2019 (Table). Several health departments reported difficulties in conducting home nursing visits and environmental investigations following identification of children with BLL above the reference value because of staffing shortages and difficulties conducting home visits. In addition, some families whose children had elevated BLLs were no longer in the listed residence.

**FIGURE:**
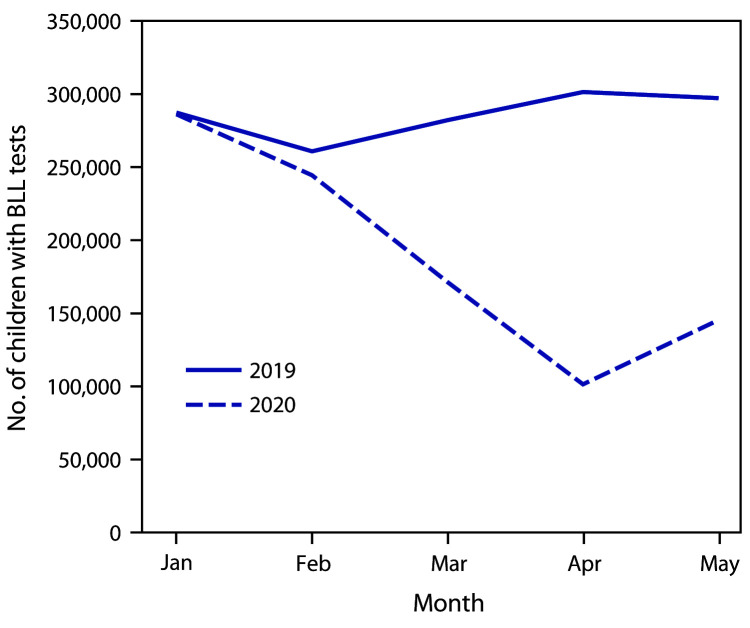
Number of children aged <6 years who received blood lead level (BLL) tests,* by month — 34 U.S. jurisdictions,**^†^** 2019–2020 * CDC requested that state and local health departments report the total number of children with BLL tests by month during January–May 2019 and January–May 2020. Data for children aged <6 years were received from 34 state and local health departments, including the District of Columbia and New York City. ^†^ Alabama, Alaska, Arizona, California, Colorado, Delaware, District of Columbia, Florida, Georgia, Hawaii, Illinois, Indiana, Iowa, Kansas, Louisiana, Maine, Maryland, Massachusetts, Michigan, Minnesota, Missouri, Nevada, New Hampshire, New Mexico, New York (excluding New York City), New York City, Ohio, Oregon, Rhode Island, Tennessee, Texas, Washington, West Virginia, and Wisconsin.

**TABLE:** Number of children aged <6 years with blood lead level (BLL) tests,* absolute change, and percentage change, by jurisdiction — 34 U.S. jurisdictions, 2019–2020

Jurisdiction	Month	No. of children tested		Absolute change, no.	% Change
		2019	2020		
**U.S. totals (for programs reporting data)**	**Jan**	**287,343**	**286,261**	**−1,082**	**−0.4**
	**Feb**	**260,861**	**244,384**	**−16,477**	**−6.3**
	**Mar**	**282,150**	**171,298**	**−110,852**	**−39.3**
	**Apr**	**301,380**	**101,388**	**−199,992**	**−66.4**
	**May**	**297,282**	**145,513**	**−151,769**	**−51.1**
**5-month totals**	**Jan–May**	**1,429,016**	**948,844**	−**480,172**	−**33.6**
Alabama	Jan	3,376	3,060	−316	−9.4
	Feb	2,914	2,219	−695	−23.9
	Mar	2,972	1,928	−1,044	−35.1
	Apr	3,563	1,328	−2,235	−62.7
	May	2,732	1,097	−1,635	−59.8
Alaska	Jan	701	561	−140	−20.0
	Feb	544	526	−18	−3.3
	Mar	659	325	−334	−50.7
	Apr	627	334	−293	−46.7
	May	581	417	−164	−28.2
Arizona	Jan	5,571	5,278	−293	−5.3
	Feb	4,701	4,501	−200	−4.3
	Mar	5,278	3,060	−2,218	−42.0
	Apr	5,470	1,819	−3,651	−66.7
	May	5,233	2,300	−2,933	−56.0
California	Jan	41,972	39,719	−2,253	−5.4
	Feb	36,939	35,170	−1,769	−4.8
	Mar	41,215	24,210	−17,005	−41.3
	Apr	43,778	12,746	−31,032	−70.9
	May	43,734	21,006	−22,728	−52.0
Colorado	Jan	1,994	1,406	−588	−29.5
	Feb	1,882	1,113	−769	−40.9
	Mar	1,826	803	−1,023	−56.0
	Apr	1,963	716	−1,247	−63.5
	May	2,060	609	−1,451	−70.4
Delaware	Jan	1,177	885	−292	−24.8
	Feb	1,068	759	−309	−28.9
	Mar	1,166	517	−649	−55.7
	Apr	1,358	126	−1,232	−90.7
	May	1,319	270	−1,049	−79.5
District of Columbia	Jan	1,411	1,109	−302	−21.4
	Feb	1,126	1,186	60	5.3
	Mar	1,357	828	−529	−39.0
	Apr	1,465	264	−1,201	−82.0
	May	1,408	567	−841	−59.7
Florida	Jan	17,839	16,928	−911	−5.1
	Feb	16,001	14,444	−1,557	−9.7
	Mar	15,165	11,667	−3,498	−23.1
	Apr	17,473	8,061	−9,412	−53.9
	May	16,993	11,385	−5,608	−33.0
Georgia	Jan	9,079	9,401	322	3.5
	Feb	8,104	7,302	−802	−9.9
	Mar	8,059	4,905	−3,154	−39.1
	Apr	8,154	3,818	−4,336	−53.2
	May	8,222	4,490	−3,732	−45.4
Hawaii	Jan	1,593	1,456	−137	−8.6
	Feb	1,378	1,315	−63	−4.6
	Mar	1,437	976	−461	−32.1
	Apr	1,627	578	−1,049	−64.5
	May	1,688	980	−708	−41.9
Illinois	Jan	17,426	18,219	793	4.6
	Feb	18,094	16,693	−1,401	−7.7
	Mar	19,265	11,326	−7,939	−41.2
	Apr	21,269	5,760	−15,509	−72.9
	May	21,014	8,700	−12,314	−58.6
Indiana	Jan	6,349	7,801	1,452	22.9
	Feb	5,920	6,586	666	11.3
	Mar	6,503	4,592	−1,911	−29.4
	Apr	6,622	2,285	−4,337	−65.5
	May	6,487	3,911	−2,576	−39.7
Iowa	Jan	5,396	5,241	−155	−2.9
	Feb	5,066	4,361	−705	−13.9
	Mar	5,616	3,567	−2,049	−36.5
	Apr	5,937	2,472	−3,465	−58.4
	May	5,969	3,277	−2,692	−45.1
Kansas	Jan	2,462	2,485	23	0.9
	Feb	2,104	2,083	−21	−1.0
	Mar	2,317	1,603	−714	−30.8
	Apr	2,670	1,163	−1,507	−56.4
	May	2,580	1,523	−1,057	−41.0
Louisiana	Jan	2,837	2,808	−29	−1.0
	Feb	2,576	2,307	−269	−10.4
	Mar	2,675	1,639	−1,036	−38.7
	Apr	2,718	1,145	−1,573	−57.9
	May	3,086	1,931	−1,155	−37.4
Maine	Jan	1,231	1,862	631	51.3
	Feb	1,013	1,420	407	40.2
	Mar	1,207	988	−219	−18.1
	Apr	1,271	766	−505	−39.7
	May	1,361	1,137	−224	−16.5
Maryland	Jan	6,300	6,153	−147	−2.3
	Feb	5,662	5,004	−658	−11.6
	Mar	6,498	3,535	−2,963	−45.6
	Apr	6,876	1,626	−5,250	−76.4
	May	7,271	2,726	−4,545	−62.5
Massachusetts	Jan	18,682	18,470	−212	−1.1
	Feb	15,917	14,996	−921	−5.8
	Mar	18,170	10,012	−8,158	−44.9
	Apr	18,868	5,594	−13,274	−70.4
	May	19,852	8,007	−11,845	−59.7
Michigan	Jan	12,006	13,224	1,218	10.1
	Feb	12,242	11,201	−1,041	−8.5
	Mar	13,421	7,181	−6,240	−46.5
	Apr	13,093	3,008	−10,085	−77.0
	May	13,400	2,266	−11,134	−83.1
Minnesota	Jan	7,551	8,040	489	6.5
	Feb	6,877	6,717	−160	−2.3
	Mar	7,180	4,803	−2,377	−33.1
	Apr	8,272	3,323	−4,949	−59.8
	May	8,096	4,198	−3,898	−48.1
Missouri	Jan	6,860	6,252	−608	−8.9
	Feb	5,881	4,851	−1,030	−17.5
	Mar	6,415	3,154	−3,261	−50.8
	Apr	6,886	1,350	−5,536	−80.4
	May	6,666	2,012	−4,654	−69.8
Nevada	Jan	663	691	28	4.2
	Feb	617	701	84	13.6
	Mar	699	409	−290	−41.5
	Apr	761	206	−555	−72.9
	May	726	279	−447	−61.6
New Hampshire	Jan	1,900	1,974	74	3.9
	Feb	1,627	1,551	−76	−4.7
	Mar	1,887	1,175	−712	−37.7
	Apr	1,932	853	−1,079	−55.8
	May	1,979	1,278	−701	−35.4
New Mexico	Jan	1,276	1,162	−114	−8.9
	Feb	1,117	881	−236	−21.1
	Mar	1,152	781	−371	−32.2
	Apr	1,365	357	−1,008	−73.8
	May	1,255	398	−857	−68.3
New York (excluding New York City)	Jan	19,553	20,385	832	4.3
	Feb	18,130	17,293	−837	−4.6
	Mar	20,463	12,771	−7,692	−37.6
	Apr	20,351	8,806	−11,545	−56.7
	May	21,633	13,088	−8,545	−39.5
New York City	Jan	26,415	27,190	775	2.9
	Feb	23,736	23,026	−710	−3.0
	Mar	26,556	13,618	−12,938	−48.7
	Apr	26,970	3,703	−23,267	−86.3
	May	27,779	10,286	−17,493	−63.0
Ohio	Jan	14,382	15,154	772	5.4
	Feb	13,440	12,865	−575	−4.3
	Mar	13,533	9,555	−3,978	−29.4
	Apr	14,878	6,377	−8,501	−57.1
	May	14,243	6,938	−7,305	−51.3
Oregon	Jan	1,817	1,843	26	1.4
	Feb	1,644	1,710	66	4.0
	Mar	1,566	1,153	−413	−26.4
	Apr	1,880	968	−912	−48.5
	May	1,707	1,330	−377	−22.1
Rhode Island	Jan	N/A	N/A	N/A	N/A
	Feb	N/A	N/A	N/A	N/A
	Mar	1,360	711	−649	−47.7
	Apr	1,425	227	−1,198	−84.1
	May	1,547	512	−1,035	−66.9
Tennessee	Jan	7,350	8,379	1,029	14.0
	Feb	6,616	7,338	722	10.9
	Mar	7,179	5,968	−1,211	−16.9
	Apr	8,256	4,629	−3,627	−43.9
	May	7,634	4,451	−3,183	−41.7
Texas	Jan	30,459	27,570	−2,889	−9.5
	Feb	26,647	24,147	−2,500	−9.4
	Mar	27,352	16,441	−10,911	−39.9
	Apr	30,569	13,107	−17,462	−57.1
	May	26,280	18,833	−7,447	−28.3
Washington	Jan	2,521	1,876	−645	−25.6
	Feb	1,802	1,701	−101	−5.6
	Mar	2,343	1,328	−1,015	−43.3
	Apr	2,200	1,010	−1,190	−54.1
	May	2,649	943	−1,706	−64.4
West Virginia	Jan	1,604	1,484	−120	−7.5
	Feb	1,569	1,328	−241	−15.4
	Mar	1,782	1,049	−733	−41.1
	Apr	1,876	624	−1,252	−66.7
	May	1,861	930	−931	−50.0
Wisconsin	Jan	7,590	8,195	605	8.0
	Feb	7,907	7,089	−818	−10.3
	Mar	7,877	4,720	−3,157	−40.1
	Apr	8,957	2,239	−6,718	−75.0
	May	8,237	3,438	−4,799	−58.3

**Abbreviation**: N/A = not available.

* CDC requested that state and local health departments report the total number of children with BLL tests by month during January–May 2019 and January–May 2020. Data for children aged <6 years were received from 34 state and local health departments, including the District of Columbia and New York City.

## Discussion

Approximately 500,000 fewer children in the reporting jurisdictions were tested for lead exposure during the first 5 months of 2020 than during the same period in 2019. Estimating from this finding, approximately 10,000 children with elevated BLL were missed because of decreased testing. Reported challenges to conducting follow-up medical visits and environmental investigations indicate delays in exposure elimination and linkage to critical services for these children. Although socioeconomic data were not collected, a disproportionate impact is anticipated among children at risk for increased lead exposure, including children from racial or ethnic minority groups, from families who have been economically or socially marginalized, and those living in older housing with lead-based paint (*1*,*3*). These groups have also been disproportionately affected by the COVID-19 pandemic (*6*,*7*). Lead testing trends among young children mirror declines in other pediatric medical services during the pandemic, including emergency department visits (*8*), well-child visits and screenings,^ §§^ and orders for childhood vaccines (*9*) and vaccination coverage (*10*). As a result of COVID-19 shelter-in-place orders and school closures, there is also concern that children spending more time in contaminated environments could have ongoing or increased exposure.

Although telemedicine and other remote service delivery strategies provide an alternative to office and clinic visits during the pandemic, in-person visits are still necessary for many essential health examinations, including BLL testing among children. During the pandemic, the American Academy of Pediatrics recommends that well-child examinations occur in person whenever possible and within the child’s medical home where continuity of care can be established.^¶¶^ CDC guidance recommends that health care providers identify children who have missed well-child visits or recommended vaccinations and contact them to schedule in-person appointments, with prioritization of infants, children aged <24 months, and school-aged children.*** It is important that health care providers ensure that all children receive lead testing, including those who missed routine BLL screening, those with prior elevated BLLs who need follow-up testing, and those with possible lead exposure. Collaborations among health departments; Special Supplementation Nutrition Program for Women, Infants, and Children programs; immunization programs; Medicaid; refugee health organizations; and other health service providers for children at risk, including outreach to parents and providers and reminders to test children at risk for lead exposure, can help ensure that these children receive needed health assessments. States and local childhood lead poisoning prevention programs can examine data from blood lead surveillance and Medicaid to identify children in need of lead testing.

The findings in this report are subject to at least two limitations. First, this report is based on preliminary surveillance data. Observed declines could be partially caused by delays in laboratory reporting and data entry backlogs. Second, use of laboratory and health department resources for COVID-19 activities could have also affected these preliminary data. However, given broader national trends for pediatric medical services, it is likely that these BLL testing data reflect actual declines.

CDC has developed guidance for conducting environmental inspections and public health home visits during the COVID-19 pandemic,^†††^ and the Health Resources and Services Administration’s Maternal and Child Health Bureau has developed guidance for conducting home health visits for young children.^§§§^ Childhood lead poisoning prevention programs can collaborate with federal and local housing and environmental health agencies to address priority housing hazards. CDC will continue to work with health departments and other partners to develop and disseminate strategies for BLL testing during the pandemic. As surveillance data become available, CDC will conduct analyses to guide decision-making and interventions toward ensuring all children receive blood lead screening and appropriate care management during the pandemic.

**Summary**

**What is already known about this topic?**

Lead can affect a young child’s ability to learn and cause other adverse health effects; no safe blood lead level (BLL) is known. Routine testing can detect elevated BLLs.

**What is added by this report?**

During January–May 2020, 34% fewer U.S. children had BLL testing compared with those during January–May 2019, with an estimated 9,603 children with elevated BLLs missed. All 34 reporting jurisdictions reported that fewer children were tested following the COVID-19 national emergency declaration in March.

**What are the implications for public health practice?**

COVID-19 has adversely affected identification of children with elevated BLLs, exposure elimination, and linkage to services. It remains important that providers ensure that young children receive appropriate lead testing and care management.

## Footnotes

* CDC uses a BLRV of 5.0 *μ*g/dL to identify children with blood lead levels that are higher than those of most children. The BLRV is based on the 97.5th percentile of the NHANES blood lead distribution in children aged 1–5 years. The current BLRV is based on NHANES data from 2007–2008 and 2009–2010.

† The U.S. Consumer Products Safety Commission banned lead-based paints for residential use in 1978.

¶ 45 C.F.R. part 46.102(l)(2), 21 C.F.R. part 56; 42 U.S.C. Sect. 241(d); 5 U.S.C. Sect. 552a; 44 U.S.C. Sect. 3501 et seq.

§§
https://www.medicaid.gov/resources-for-states/downloads/medicaid-chip-beneficiaries-18-under-COVID-19-snapshot-data.pdf

¶¶
https://services.aap.org/en/pages/2019-novel-coronavirus-covid-19-infections/clinical-guidance/

*** Developmental surveillance and early childhood screenings, including developmental and autism screening, should continue along with referrals for early intervention services and further evaluation if concerns are identified. https://www.cdc.gov/coronavirus/2019-ncov/hcp/pediatric-hcp.html

†††
https://www.cdc.gov/coronavirus/2019-ncov/community/organizations/ph-inspectors-employees.html

§§§
https://mchb.hrsa.gov/Home-Visiting-Information-During-COVID-19
